# Hydrodynamic Cavitation: A Promising Technology for Industrial-Scale Synthesis of Nanomaterials

**DOI:** 10.3389/fchem.2020.00259

**Published:** 2020-04-15

**Authors:** Xun Sun, Songying Chen, Jingting Liu, Shan Zhao, Joon Yong Yoon

**Affiliations:** ^1^Key Laboratory of High Efficiency and Clean Mechanical Manufacture, Ministry of Education, National Demonstration Center for Experimental Mechanical Engineering Education at Shandong University, School of Mechanical Engineering, Shandong University, Jinan, China; ^2^Shandong Key Laboratory of Water Pollution Control and Resource Reuse, School of Environmental Science and Engineering, Shandong University, Qingdao, China; ^3^Department of Mechanical Engineering, Hanyang University, Ansan, South Korea

**Keywords:** sonochemistry, synthesis of nanomaterials, hydrodynamic cavitation, hydrodynamic cavitation reactor, application potentiality

## Abstract

One of the most challenging issues for the large-scale application of nanomaterials, especially nanocarbons, is the lack of industrial synthetic methods. Sonochemistry, which creates an extreme condition of high pressure and temperature, has been thereby applied for synthesizing a wide variety of unusual nanostructured materials. Hydrodynamic cavitation (HC), characterized by high effectiveness, good scalability, and synergistic effect with other physical and chemical methods, has emerged as the promising sonochemistry technology for industrial-scale applications. Recently, it was reported that HC can not only significantly enhance the performance of biochar, but also preserve or improve the respective chemical composition. Moreover, the economic efficiency was found to be at least one order of magnitude higher than that of conventional methods. Due to the great potential of HC in the industrial-scale synthesis of nanomaterials, the present perspective focuses on the mechanism of sonochemistry, advances in HC applications, and development of hydrodynamic cavitation reactors, which is supposed to contribute to the fundamental understanding of this novel technology.

## Introduction

The concept of nanoscience and nanotechnology was first proposed by Richard Feynman in 1959 (Feynman, 1992). Until 1974, the term *nanotechnology* (Taniguchi, 1974) was introduced by Nario Taniguchi during a scientific conference. With the help of the scanning tunneling microscope, which was invented by Gerd Binnig and Heinrich Rohrer in 1981 (Binnig and Rohrer, 1987), the modern nanotechnology has been developing rapidly since then. Recent advances in nanomaterials have significantly influenced various fields, e.g., material science, medical science, environmental science, magnetics, mechanics, and optics. Because the synthesis method largely determines the physical properties and applications of nanomaterials, developing new methods to design appropriate synthetic routes has been the research hotspot (Wang et al., 2019; Xu et al., 2019). Among the diverse physical and chemical synthesis methods, sonochemistry method has been considered one of the most powerful tools for synthesizing nanomaterials (Bang and Suslick, 2010). Sonochemistry can be effectively induced by ultrasounds (i.e., acoustic cavitation, AC) or local pressure drop (i.e., hydrodynamic cavitation, HC). AC has been utilized to achieve a wide variety of unusual nanostructured materials at laboratory scale, e.g., metals, metal oxides, metal chalcogenides and carbides, carbon, protein, and polymer. However, further application in industrial-scale may be considerably difficult as the issues of scale-up and energy efficiency (Gagol et al., 2018). Recently, HC, which has emerged as the promising technology for various industrial-scale applications, was found to be an effective tool for synthesis of nanomaterials through mechanisms similar to that of AC. The present paper aims to discuss the mechanism of sonochemistry, recent advances in the HC technology development, and its application perspective for synthesis of nanomaterials.

## Sonochemistry

Unlike traditional energy sources such as heat, light, or ionizing radiation that are required for chemical reactions to proceed, sonochemistry is a unique energy-matter interaction that occurs without direct interaction with molecular species (Thompson and Doraiswamy, 1999). Sonochemistry derives principally from cavitation which is a rapid phase-change phenomenon in liquids, consisting of growth and collapse of cavitation nuclei during an extremely short period (Suslick, 1990). When the bubble collapses, a huge amount of energy, on the order of 1–10^18^ kW/m^3^, can be released into surrounding liquids (Gogate et al., 2006). The released energy can be divided into three forms (Sun et al., 2018b).

Mechanical effect: Generation of shock waves with propagation velocities of 2,000 m/s in average (Holzfuss et al., 1998), micro-jets with high water-hammer pressure (450 MPa; Vogel et al., 1989) and velocity (over 120 m/s; Benjamin and Ellis, 1966; Lauterborn and Bolle, 1975; Shima et al., 1981), and high shear stresses (as high as 3.5 kPa; Dijkink and Ohl, 2008).

Thermal effect: Production of local hot spots (2,000–6,000 K, depending on the distance; Hart et al., 1990; Flint and Suslick, 1991; Didenko et al., 1999) with heating/cooling rates >10^10^ K/s (Suslick et al., 1986).

Chemical effect: Formation of highly active hydroxyl radicals, with an oxidation potential of 2.8 V, by the sonolysis of water molecules (Arrojo et al., 2007; Kuppa and Moholkar, 2010).

The combination of the above three effects creates extreme conditions for synthesis of nanomaterials at ambient conditions (e.g., room temperature and atmospheric pressure) and sometimes even without the utilization of catalysts. Taking synthesizing nanostructured carbon materials as an example, sonochemistry effect can not only enhance the reactions which can lead to exotic carbon nanostructures (Sun et al., 2002), but also induce dramatic morphology changes in pre-synthesized carbon materials (Viculis et al., 2003). However, due to the characteristics of AC, the energy density rapidly attenuates with increasing distance from the ultrasonic horn and disappears at a distance of as low as 2–5 cm (Gagol et al., 2019). Therefore, to achieve the desired energy density, a number of ultrasonic horns have to be closely arranged in an AC reactor. This inherent feature of AC causes the equipment prices and operational costs rise rapidly with the scale (Gagol et al., 2018), which indicates that AC is not suitable for industrial-scale synthesis of nanomaterials. On the other hand, HC, which can effectively induce sonochemistry by utilizing a mechanical approach, has the ability to overcome the inherent defect of AC.

## Hydrodynamic Cavitation

Unlike AC, which is generated by applying ultrasound waves with a cyclic succession of expansion (rarefaction) and compression phases on a liquid (Vajnhandl and Majcen Le Marechal, 2005), HC is induced by static pressure drops of the flowing liquid. When the flow passes through constricted parts or irregular geometries, the flow velocity increases and then, a decrease in static pressure can be caused. Once the pressure falls below the local saturated vapor pressure, cavitation nuclei existing in water begin to grow because their internal pressures become greater than the surface tension. When the flow pressure recovers, the growing nuclei become unstable and collapse (Yan and Thorpe, 1990). The working principle of a typical HC system (Venturi) is shown in **Figure 2A** (Šarc et al., 2018). The liquids in the reservoir are pumped to the Venturi section, cavitation phenomenon occurs in the diffusion part of the Venturi, and then, the liquids are sent back to the reservoir. The above process will continue for a period of time until the satisfactory treatment effect is obtained.

**Figure 1:**
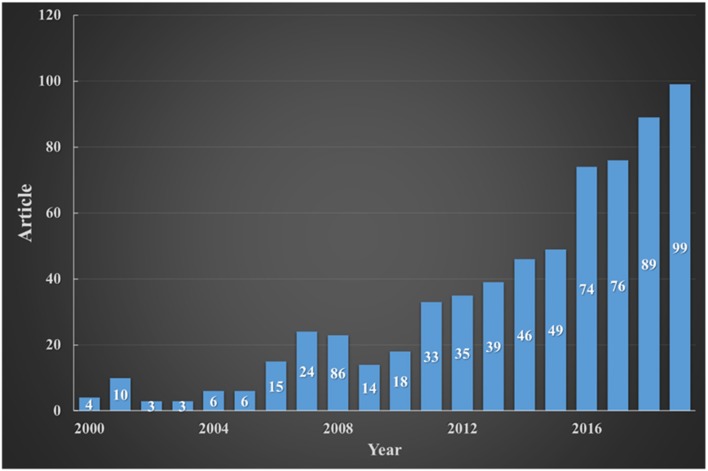
Articles about HC application from 2000 to 2019 (based on Google Scholar available on 25.2.2020).

The exploration of HC began at the beginning of the 20th century as a negative consequence of erosion damage. In 1912, Silberrad reported that cavitation was associated with severe destructive damage to the propellers of the great ocean liners *Lusitania* and *Mauretania* (Silberrad, 1912). Since then, the researchers have been focusing on the negative effect of cavitation, e.g., performance losses of various fluid machinery, noise, and erosion damage (Rahmeyer, 1981; Sun et al., 2017b). On the other hand, Save et al. (1994) presented the first case study for microbial cell disruption by utilizing HC in 1994. After that, the applications of HC have begun to attract attention in a wide variety of areas, especially in the last few years (Figure 1). Nowadays, researchers have found that HC can be an effective tool for a number of chemical, biological, and other types of applications, e.g., microbial inactivation [bacteria (Mane et al., 2020), algae (Waghmare et al., 2019), virus (Kosel et al., 2017)], the removal of organic compounds (acids Choi et al., 2019, antibiotics (Tao et al., 2018), pesticides (Panda and Manickam, 2019), dyes Yi et al., 2018, pharmaceuticals (Rajoriya et al., 2019), fuel (Torabi Angaji and Ghiaee, 2015), phenols Chakinala et al., 2008, etc.,) decomposition of waste-activated sludge (WAS) (Nabi et al., 2019), depolymerization (Prajapat and Gogate, 2019), denitrification (Song et al., 2019), desulfurization (Gagol et al., 2019), fibrillation (Kosel et al., 2019), intensification of biogas production (Zielinski et al., 2019), biofuel synthesis (Chipurici et al., 2019), liposome destruction (Pandur et al., 2020), catalyst slurry preparation (Kuroki et al., 2019), flotation (Ross et al., 2019), food processing (Terán Hilares et al., 2019), surface finishing (Nagalingam et al., 2019), viscosity reduction (Gregersen et al., 2019), residual stress relief, cleaning, and emulsification (Wu et al., 2019).

Until last year, the first study on the nanomaterial preparation by utilizing HC was reported by Albanese et al. (2019) who utilized HC to enhance the surface area of biochar by as much as 120%, while preserving or improving the respective chemical composition. The increases in functionality and porosity of the biochar were contributed to the effect of the physical impact and oxidation (hydroxyl radicals) of HC. Moreover, the economic efficiency was found to be at least one order of magnitude higher than that of the conventional method, which demonstrates that HC can be an effective alternative approach for synthesis of nanomaterials. HC phenomenon is induced by hydrodynamic cavitation reactor (HCR), i.e., the container for HC reaction, therefore, the HC generation efficiency of HCR determines the treatment effect, economy, and applicability of HC technology. To accelerate the development of HC-based synthetic strategies of industrial-scale production of nanomaterials, the most important thing is to develop new-family HCRs. Therefore, we further make a simple overview of the development of HCR in order to give readership more clearer understandings about this new field.

## Development of HCR

**Figure 2:**
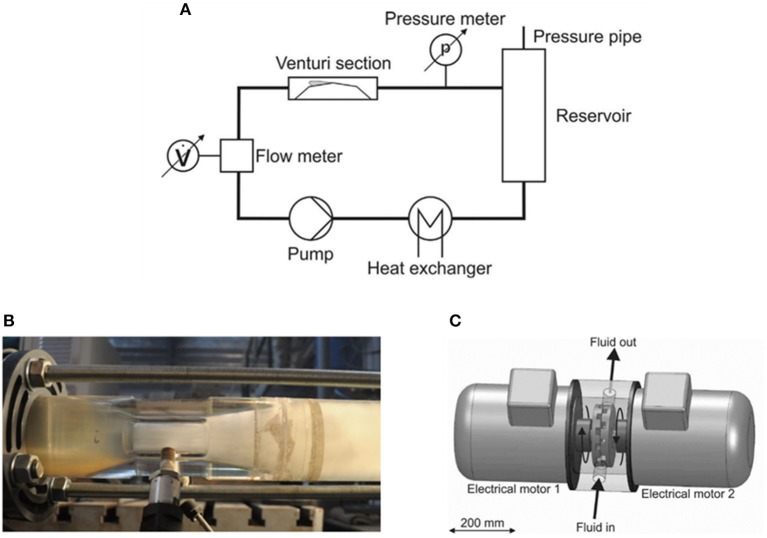
Schematic diagram of representative HC working principle **(A)** Šarc et al. (2018) and HCRs, **(B)** Venturi, and **(C)** rotor-stator type (Zupanc et al., 2014).

HCRs can be generally categorized into two types based on their operational mechanism: non-rotational and rotational. In common conventional HCRs [CHCRs, *e.g*., Venturi type (Figure 2B; Jančula et al., 2014) and orifice type], low-pressure separation region can be formed when the fluid passes through the contractive portion where the static pressure is converted to kinetic energy (Merzkirch et al., 2015). To understand the mechanisms of HC treatment, CHCRs have been widely utilized in laboratory scale in the previous studies, because of the advantages they offer in simple design, lack of moving parts, and ease of manufacture and use (Dular et al., 2016). Moreover, some researchers found that CHCRs can be applied to real industrial applications (Hirooka et al., 2009).

Recently, a few rotor-stator type HCRs (R-S HCRs) have been introduced in the literature. R-S HCRs utilize a circular disk or cylinder with numerous gaps to generate cavitation (Figure 2C; Zupanc et al., 2014). Due to the viscosity, the fluid is driven by the rotor and the flow direction is identical to the rotational direction. The flow punches the back edge of the gap and forms separation region with low pressure. Cavitation bubbles can occur when the rotational speed reaches the critical value. The results from the corresponding research indicated the effectiveness of the treatment, economic efficiency which is far beyond those of traditional devices in the removal of microorganism (Milly et al., 2007, 2008; Šarc et al., 2018; Sun et al., 2018a,b; Maršálek et al., 2020), WAS treatment (Petkovšek et al., 2015; Kim et al., 2019, 2020; SeŽun et al., 2019), organic wastewater treatment (Badve et al., 2013; Zupanc et al., 2014), biofuel synthesis (Mohod et al., 2017; Chipurici et al., 2019), fibrillation (Kosel et al., 2019), intensification of biogas production (Patil et al., 2016), and delignification (Badve et al., 2014), etc., even without geometrical optimization or in scaled-up application. In addition, due to the extreme conditions and the hydroxyl radicals produced by HC, effective synergic effects between HC and heating, AC (Sun et al., 2018a), various oxidants (Saharan et al., 2011), photocatalyst (Wang et al., 2011), photolysis (Zupanc et al., 2014), and electrochemical (Wang et al., 2010) have been proved. More importantly, it is worth noting that R-S HCRs show promising scalability (Joshi and Gogate, 2019), and their performance can be easily improved by scaling up the dimensions, which was confirmed by Sun et al. (2018a) who found that when the rotor of the HCR doubled in size (290–590 mm), heat generation and thermal efficiency increased from 48 to 200 MJ/h and 82 to 91%, respectively.

So far, HC technology has not been widely utilized in industrial applications around the world, even though it has been investigated and developed for nearly 30 years. Most of the existing research relates to applications, the characteristics of HCRs have been rarely focused, which largely influences the development and application of HC technology. Even though a few researchers have made important contributions in theoretical (Sarvothaman et al., 2019), computational (Badve et al., 2015), and experimental (Zhang et al., 2018) aspects of HCRs, their cavitation generation mechanism, internal flow fields, external characteristics, and scale-up law are not well understood by utilizing experimental flow visualization, particle image velocimetry, and computational fluid dynamic methods, especially for the R-S HCRs. More importantly, the universal research and design methods (e.g., the theoretical and numerical methods for design the rotor, stator, and flow path, scale-up law, and optimization method) for HCRs have not been established yet. The investigations on the internal fluid field, geometrical optimizations, numerical simulation methods, dimensional analyses, and similarity laws for the external characteristics, etc., are required in future.

## Conclusion and Perspective

The present paper illustrated the mechanism of sonochemistry, advances in HC applications, and development of hydrodynamic cavitation reactors, with the aim to contribute to the fundamental understanding of this novel technology. With the mechanism similar to that of AC, HC technology appears to be an effective sonochemistry means for synthesizing nanomaterials in industrial-scale due to its good scalability. The development and application of HC synthetic method will be a real challenge because of its highly interdisciplinary (related to sonochemistry, fluid dynamics, material science, and mechanical engineering). However, significant progress in this technology will lead to the considerable promotion of the industrialization of nanomaterials. Several challenges and research directions that can be considered are outlined below:

- To understand the HC mechanism, it is necessary to apply HC to the preparation of various types of nanomaterials, including metals, alloys, oxides, sulfides, carbides, carbons, polymers, and biomaterials.
- Studying the synergistic effects between HC and traditional synthetic methods on the structure and performance of nanomaterials.
- Developing appropriate CFD methods to reveal the cavitation generation mechanism and design new HCRs.
- Optimizing the geometrical structure of HCRs by advanced algorithms (Sun et al., 2017a; Sun and Yoon, 2018).
- Establishing the universal research and design methods for HCRs.

## Data Availability Statement

All datasets generated for this study are included in the article/supplementary material.

## Author Contributions

XS, JY, and SC contributed conception of the study. XS produced and wrote the article. JL and SZ edited the article.

### Conflict of Interest

The authors declare that the research was conducted in the absence of any commercial or financial relationships that could be construed as a potential conflict of interest.
